# Association Between Markers of Structural Racism and Mass Shooting Events in Major US Cities

**DOI:** 10.1001/jamasurg.2023.2846

**Published:** 2023-07-19

**Authors:** Michael Ghio, John Tyler Simpson, Ayman Ali, Julia M. Fleckman, Katherine P. Theall, Joseph I. Constans, Danielle Tatum, Patrick R. McGrew, Juan Duchesne, Sharven Taghavi

**Affiliations:** 1 Department of Surgery, Tulane University School of Medicine, New Orleans, Louisiana; 2 Tulane University School of Public Health and Tropical Medicine, New Orleans, Louisiana; 3 Tulane University School of Science & Engineering, New Orleans, Louisiana; 4 University Medical Center, New Orleans, Louisiana

## Abstract

**Importance:**

The root cause of mass shooting events (MSEs) and the populations most affected by them are poorly understood.

**Objective:**

To examine the association between structural racism and mass shootings in major metropolitan cities in the United States.

**Design, Setting, and Participants:**

This cross-sectional study of MSEs in the 51 largest metropolitan statistical areas (MSAs) in the United States analyzes population-based data from 2015 to 2019 and the Gun Violence Archive. The data analysis was performed from February 2021 to January 2022.

**Exposure:**

Shooting event where 4 or more people not including the shooter were injured or killed.

**Main Outcome and Measures:**

MSE incidence and markers of structural racism from demographic data, Gini income coefficient, Black-White segregation index, and violent crime rate.

**Results:**

There were 865 MSEs across all 51 MSAs from 2015 to 2019 with a total of 3968 injuries and 828 fatalities. Higher segregation index (ρ = 0.46, *P* = .003) was associated with MSE incidence (adjusted per 100 000 population) using Spearman ρ analysis. Percentage of the MSA population comprising Black individuals (ρ = 0.76, *P* < .001), children in a single-parent household (ρ = 0.44, *P* < .001), and violent crime rate (ρ = 0.34, *P* = .03) were other variables associated with MSEs. On linear regression, structural racism, as measured by percentage of the MSA population comprising Black individuals, was associated with MSEs (β = 0.10; 95% CI, 0.05 to 0.14; *P* < .001). Segregation index (β = 0.02, 95% CI, −0.03 to 0.06; *P* = .53), children in a single-parent household (β = −0.04, 95% CI, −0.11 to 0.04; *P* = .28), and Gini income coefficient (β = −1.02; 95% CI, −11.97 to 9.93; *P* = .93) were not associated with MSEs on linear regression.

**Conclusions and Relevance:**

This study found that major US cities with higher populations of Black individuals are more likely to be affected by MSEs, suggesting that structural racism may have a role in their incidence. Public health initiatives aiming to prevent MSEs should target factors associated with structural racism to address gun violence.

## Key Points

**Question:**

Who are the victims of mass shooting events, and are there associated risk factors?

**Findings:**

This cross-sectional study examining approximately 900 mass shooting events found that Black individuals were disproportionately affected by mass shooting events.

**Meaning:**

There is evidence that structural racism may play a role in mass shooting events.

## Teaser

This cross-sectional study examines data from the Gun Violence Archive for mass shooting events that occurred from 2015 to 2019 in the 51 largest metropolitan statistical areas in the United States to determine associations with markers of structural racism.

## Introduction

Gun violence in the United States has steadily increased over the last several decades. The Centers for Disease Control and Prevention reports that firearms were used in 81% of all homicides and 55% of all suicides in 2021.^1^ Research shows that racial and ethnic minority communities are disproportionately affected by firearm violence and mortality as compared with White communities. Although Black and African American individuals represent only 13.6% of the US population, they are disproportionately counted among the victims of firearm-related homicides.^2^ In 2021, 58% of firearm homicide victims were reported to be Black while 37% were White.^2^

Accompanying the overall increase in firearm violence is a considerable rise in the number of mass shooting events (MSEs). While there is not consensus on what constitutes an MSE, a commonly accepted definition used by many databases, including the Gun Violence Archive (GVA), is a shooting incident in which 4 or more individuals are shot and wounded or killed.^3^ From 2014 to 2020, there has been an increase in mass shootings, from 269 events in 2014 to 611 MSEs in 2020, which represents a 127% increase over a 6-year period.^4^ In 2022, there were a staggering 649 MSEs, the most in 1 year in the history of the United States.^4^ These 649 MSEs accounted for 3.34% of all firearm-related mortalities and 7.00% of firearm-related injuries.^4^

Community gun violence has been linked to structural racism, which has been defined as “the normalized and legitimized range of policies, practices, and attitudes that routinely produce cumulative and chronic adverse outcomes for people of color.”^5,6,7^ Firearm injuries are more likely to occur in low-income areas, and Black individuals make up the majority of those injured or killed in shootings.^8^ However, the way in which measures of structural racism correlate with MSEs has not been elucidated. In this study, we sought to examine the association between structural racism and MSEs in metropolitan cities in the United States. Our hypothesis was that MSEs in metropolitan areas would predominately be a consequence of structural racism, affecting cities with a higher Black population.

## Methods

The GVA was queried between 2015 and 2019, and incidents reported (via incident address) in the 51 largest US metropolitan statistical areas (MSAs) were examined. These 51 MSAs are frequently used as a basis for data in gun violence publications. The GVA is a publicly available, not-for-profit database that records gun violence incidents collected from more than 7500 sources across the country.^3^ The range 2015-2019 was selected for analysis because this is also the date range of the Black-White segregation index, a publicly available analysis from William H. Frey and the Brookings Institute.^9^ Because these data are public and deidentified, our study was ruled exempt from institutional review board approval by the Tulane University School of Medicine institutional review board. The GVA defined an MSE as a firearm incident in which 4 or more individuals other than the shooter were injured in a single event.^3^

In this study, measures of structural racism were examined, including the Black-White segregation index and the percentage of the population that is Black. Social determinants of health we examined included poverty rate measures, educational attainment, and crime rate. The Black-White segregation index varies from values of 0, representing complete integration, to 100, complete segregation.^9^ This is estimated by measuring the index of dissimilarity. Dissimilarity is determined through examination of the distribution of Black individuals across a neighborhood compared with that of their White peers.^9^

Demographic data (2015-2019) and Gini coefficients (2019) were obtained from the US Census Bureau and the US Department of Education. The Gini index is a measure of income inequality that ranges from 0 (perfect equality with everyone receiving a fair share of income) to 1 (perfect inequality, where 1 group or population receives a disproportionately large share of income).^10^ Poverty rate percentage (2019) was obtained from the US Census Bureau, which sets poverty thresholds and communicates these data per MSA. Educational attainment, the rate of high school graduation and percentage of the population with a bachelor’s degree, was also obtained from the US Census Bureau for each MSA. State gun law data from 2019 were obtained through the Giffords Law Center, which provided a letter grade for each state’s laws from A to F (from most safe to least safe, respectively).^11^

To examine the association between MSEs and other variables, Spearman ρ and linear regression were performed. In these analyses, MSE incidence per 100 000 was treated as a continuous variable. The model covariates considered were segregation index, unemployment rate, poverty rate, high school graduation rate, population 25 years and older with a bachelor’s degree, percentage of the MSA comprising Black individuals, children in a single-parent household, violent crime rate, and Gini coefficient.

Using the Shapiro-Wilk test, normality of residuals was confirmed. Overall fit of the linear regression model was calculated with an *R^2^*, and statistical significance was determined by using a 2-sided test with a level of *P* < .05. Our primary outcome of interest was MSE incidence and its association with measures of structural racism. In the regression of overall rate of MSE, after examination of all covariates listed above for colinearity, final model covariates included percentage of the MSA population comprising Black individuals, segregation index, and Gini coefficient. Statistical analysis was completed using SPSS version 28.0.1.1 (IBM) and performed from February 2021 to January 2022.

## Results

Overall, there were 865 MSEs in the GVA (Table 1). Only 1 MSA had no MSEs in the study period (Providence, Rhode Island), so it was excluded from analysis. There were in total 3968 injuries, with a mean number of 4.78 injuries per MSE, and 828 fatalities, representing a mean of 1.12 fatalities per MSE. Chicago had the greatest number of MSEs, with a total of 141 events, 97 fatalities (0.69/event), and 583 injuries (4.13/event). Las Vegas had the greatest number of individuals killed (5.92/event) and injured (39.75/event) with only a total of 12 MSEs; the 2017 shooting that injured 413 persons and killed 60 persons skewed these data significantly.

**Table 1.** Demographic Characteristics of 51 MSAs

MSA principal city	MSA total population, No.	Black population in MSA, %	Segregation index^a^	State firearm legislation grade^b^	Total No. of MSEs	Mean No. of individuals affected per MSE	
						Injured	Killed
Atlanta, GA	6 089 815	33	58.8	F	18	4.33	0.56
Austin, TX	2 283 371	7	49.1	F	5	3.80	0.60
Baltimore, MD	2 844 510	28	63.9	A−	47	4.04	0.57
Birmingham, AL	1 115 289	30	64.5	F	10	4.30	0.50
Boston, MA	4 941 631	7	65.0	A−	8	4.13	0.38
Buffalo, NY	1 166 902	11	75.2	A−	9	3.67	0.78
Charlotte, NC	2 660 329	21	53.1	D	11	3.82	0.55
Chicago, IL	9 618 502	16	75.3	A−	141	4.13	0.69
Cincinnati, OH	2 256 884	11	67.3	D	12	5.25	1.00
Cleveland, OH	2 088 251	19	72.9	D	22	3.82	1.10
Columbus, OH	2 138 926	16	62.4	D	11	3.91	1.00
Dallas, TX	7 637 387	15	56.8	F	12	4.00	1.33
Denver, CO	2 963 821	5	62.4	C+	9	3.56	0.67
Detroit, MI	4 392 041	21	73.7	C	25	3.80	1.08
Grand Rapids, MI	1 087 592	7	65.0	C	1	8.00	1.00
Hartford, CT	1 213 531	11	65.7	A−	6	3.67	0.50
Houston, TX	7 122 240	17	60.3	F	32	3.50	1.50
Indianapolis, IN	2 111 040	15	64.4	D−	16	3.44	0.94
Jacksonville, FL	1 605 848	20	53.7	C−	19	3.84	1.26
Kansas City, MO	2 192 035	12	59.5	F	12	4.25	0.58
Las Vegas, NV	2 265 461	11	39.5	C+	12	39.75	5.92
Los Angeles, CA	13 200 988	6	66.8	A	30	4.03	0.83
Louisville, KY	1 285 439	13	57.7	F	14	4.50	0.57
Memphis, TN	1 337 779	47	60.1	D−	29	4.14	0.52
Miami, FL	6 138 333	19	63.9	C−	22	4.18	0.73
Milwaukee, WI	1 574 731	16	79.8	C−	9	3.67	0.56
Minneapolis, MN	3 690 261	9	55.2	C+	14	4.50	0.21
Nashville, TN	1 989 519	14	54.2	D−	7	4.00	0.71
New Orleans, LA	1 271 845	33	63.5	F	34	4.62	0.97
New York, NY	20 140 470	15	76.1	A−	37	4.59	0.38
Oklahoma City, OK	1 425 695	10	51.4	F	8	3.5	0.75
Orlando, FL	2 673 376	15	49.8	C−	16	7.06	4.38
Philadelphia, PA	6 245 051	19	67.0	C+	46	4.09	0.74
Phoenix, AZ	4 845 832	5	49.2	F	11	3.91	1.64
Pittsburgh, PA	2 370 930	7	66.1	C+	12	3.67	1.83
Portland, OR	2 512 859	3	51.3	C+	2	4.50	0
Providence, RI	1 676 579	4	58.1	B+	0	0	0
Raleigh, NC	1 413 982	18	42.2	D	2	3.00	1.00
Richmond, VA	1 314 434	28	52.1	D	7	3.86	0.43
Riverside, CA	4 599 839	7	46.4	A	3	5.33	0
Rochester, NY	1 090 135	10	65.1	A−	5	4.60	1.20
Sacramento, CA	2 397 382	7	57.2	A	12	3.75	0.67
San Antonio, TX	2 558 143	6	49.1	F	15	3.93	0.53
San Diego, CA	3 298 634	4	52.2	A	7	3.57	1.57
San Francisco, CA	4 749 008	7	61.0	A	11	3.64	1.18
Seattle, WA	4 018 762	6	51.6	B+	4	3.50	1.00
St. Louis, MO	2 820 253	17	71.7	F	41	3.37	1.05
Tampa, FL	3 175 275	11	54.8	C−	6	3.83	1.00
Tucson, AZ	1 043 433	3	43.5	F	1	5.0	1.00
Virginia Beach, VA	1 799 674	28	47.3	D	4	4.25	3.50
Washington, DC	6 385 162	24	61.3	NA	18	4.39	0.44

Abbreviations: MSA, metropolitan statistical area; MSE, mass shooting event; NA, not available.

^a^ Higher segregation index values are associated with a higher geographic degree of Black-White segregation.

^b^ Letter grades A through F for state gun laws were obtained from the Giffords Law Center and indicate most safe to least safe, respectively.

The cities with the highest segregation indexes included Milwaukee, Wisconsin (79.8), New York, New York (76.1), and Detroit, Michigan (73.7). The MSAs with the lowest SI scores included Las Vegas, Nevada (39.5), Raleigh, North Carolina (42.2), and Tucson, Arizona (43.5). With regards to state firearm grades, 12 MSAs were in states with a grade of A− or A (316 total MSE, mean 26.33/MSA); 2 of B+ (4 total MSE, mean 2/MSA); 13 of C−, C, or C+ (193 total MSE, mean 14.85/MSA); 10 of D− or D (121 total MSE, mean 12.1/MSA); and 13 of F (213 total MSE, mean 16.38/MSA). Washington, DC, does not have a grade associated with it.

As seen in Table 2, unemployment rate, principal city poverty rate, Gini index, high school graduation rate, and percentage of the population 25 years and older with a bachelor’s degree are listed for each of the 51 MSAs. The MSAs with the highest unemployment rate included Baltimore, Maryland (8.7%) along with Philadelphia, Pennsylvania; Buffalo, New York; and Washington, DC, which are all tied at 5.0%. The MSAs with the lowest unemployment rates included Miami, Florida (1.8%), San Francisco, California (2.1%), and Denver, Colorado (2.3%). The Gini coefficient was highest (greatest income inequality) in Cleveland, Ohio (0.54), New York, New York (0.52), and New Orleans, Louisiana (0.50). The Gini coefficient was the lowest (least income inequality) in Grand Rapids, Michigan (0.43), Austin, Texas (0.44), and Miami, Florida (0.44). The poverty rate was highest in Tucson, Arizona (19.0%), New Orleans, Louisiana (18.4%), and Las Vegas, Nevada (15.2%) and lowest in Denver, Colorado (8.4%), Seattle, Washington (8.6%), and Washington, DC (8.7%).

**Table 2.** Social Elements of 51 MSAs

MSA principal city	Unemployment rate, %	Principal city poverty rate, %	Gini index^a^	High school graduation rate, %	Population age ≥25 y with bachelor’s degree, %
Atlanta, GA	3.5	11.6	0.47	90.9	42.2
Austin, TX	3.6	10.3	0.44	91.9	50.0
Baltimore, MD	8.7	10.7	0.46	92.3	42.9
Birmingham, AL	4.1	14.3	0.49	90.1	34.4
Boston, MA	3.0	9.3	0.48	92.1	51.1
Buffalo, NY	5.0	13.5	0.46	92.5	36.9
Charlotte, NC	3.4	10.7	0.48	90.3	39.3
Chicago, IL	3.1	11.5	0.48	89.8	40.6
Cincinnati, OH	3.7	12.1	0.47	91.9	36.5
Cleveland, OH	4.2	13.0	0.54	92.1	34.1
Columbus, OH	3.7	12.8	0.45	92.0	35.3
Dallas, TX	3.1	11.0	0.46	87.1	38.3
Denver, CO	2.3	8.4	0.45	91.9	47.8
Detroit, MI	4.1	13.4	0.48	91.3	34.2
Grand Rapids, MI	2.9	9.4	0.43	91.9	35.8
Hartford, CT	3.5	10.1	0.47	91.8	41.4
Houston, TX	3.6	14.1	0.48	84.5	35.8
Indianapolis, IN	3.0	10.6	0.47	91.8	37.8
Jacksonville, FL	3.3	12.2	0.47	92.2	35.3
Kansas City, MO	3.3	9.7	0.45	93.4	39.5
Las Vegas, NV	3.7	15.2	0.49	85.4	27.6
Los Angeles, CA	4.3	13.1	0.49	82.1	37.1
Louisville, KY	4.1	10.6	0.46	91.7	39.3
Memphis, TN	4.2	16.9	0.49	89.5	31.0
Miami, FL	1.8	13.3	0.44	87.0	35.3
Milwaukee, WI	3.5	12.4	0.48	92.6	39.2
Minneapolis, MN	2.8	8.0	0.45	94.5	44.7
Nashville, TN	2.5	10.6	0.46	91.9	40.2
New Orleans, LA	4.3	18.4	0.50	88.1	33.6
New York, NY	3.7	12.9	0.51	87.9	43.6
Oklahoma City, OK	2.8	14.4	0.46	89.5	32.5
Orlando, FL	2.8	12.9	0.46	91.3	35.2
Philadelphia, PA	5.0	12.3	0.48	91.9	41.9
Phoenix, AZ	4.7	11.1	0.46	89.3	34.6
Pittsburgh, PA	4.2	11.2	0.47	94.7	38.1
Portland, OR	2.5	10.0	0.44	93.1	42.2
Providence, RI	3.3	11.6	0.46	87.7	34.1
Raleigh, NC	3.1	10.1	0.45	92.7	50.7
Richmond, VA	2.6	10.5	0.47	91.2	40.2
Riverside, CA	4.1	12.3	0.45	82.8	23.7
Rochester, NY	3.7	12.3	0.46	91.3	37.5
Sacramento, CA	3.2	11.8	0.46	89.6	36.4
San Antonio, TX	3.1	13.4	0.45	86.5	31.4
San Diego, CA	3.1	10.6	0.46	88.4	42.0
San Francisco, CA	2.1	9.0	0.48	89.6	51.8
Seattle, WA	2.7	8.6	0.46	93.6	46.9
St. Louis, MO	3.0	10.6	0.46	93.0	37.1
Tampa, FL	2.8	13.0	0.48	90.9	34.5
Tucson, AZ	4.2	19.0	0.47	88.1	29.1
Virginia Beach, VA	2.7	10.7	0.44	93.0	36.1
Washington, DC	5.0	8.7	0.45	91.9	53.4

Abbreviation: MSA, metropolitan statistical area.

^a^ Measure of income inequality that ranges from 0 (perfect equality with everyone receiving a fair share of income) to 1 (perfect inequality, where 1 group or population receives a disproportionately large share of income).

### Spearman ρ Correlation Matrix

There were several significant associations as evidenced by Spearman ρ correlation matrix. MSEs were associated with higher segregation index (ρ = 0.46, *P* = .003) (Figure). Other variables associated with MSEs included percentage of the population comprising Black individuals (ρ = 0.76, *P* < .001), children in a single-parent household (ρ = 0.44, *P* = .008), Gini coefficient (ρ = 0.29, *P* = .01), and violent crime rate (ρ = 0.34, *P* = .03). There were no inverse associations between MSE incidence and the variables.

**Figure.**
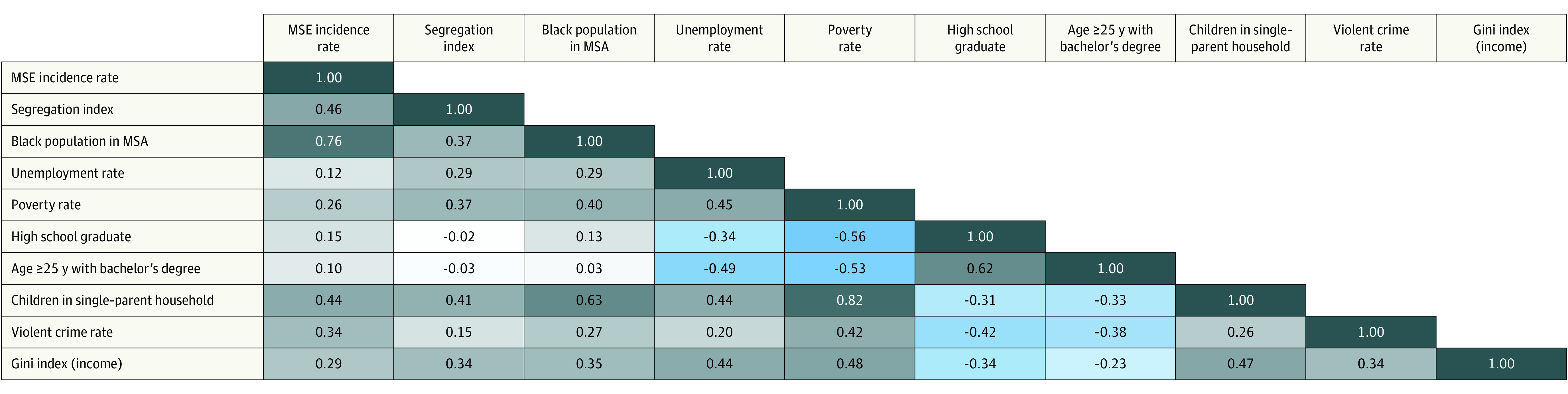
Spearman ρ Correlation Matrix for Variables Analyzed for 51 Metropolitan Statistical Areas (MSAs) Mass shooting event (MSE) incidence rate per 100 000 population was associated with segregation index (*P* = .003), percentage of MSA population comprising Black individuals (*P* < .001), children in a single-parent household (*P* = .008), Gini coefficient (*P* = .01), and violent crime rate (*P* = .03). Lighter to darker shading represents the intensity of the relationship from 0 to 1; gray boxes indicate a positive relationship and blue a negative relationship.

### Multivariate Analysis

Linear associations were observed between MSE incidence and each independent variable. Shapiro-Wilk testing for normality of the residuals was not statistically significant. The final model had an *R^2^* of 0.55 (Table 3). Structural racism, as measured by the percentage of the population that is Black, was associated with MSE incidence (β = 0.10; 95% CI, 0.05 to 0.14; *P* < .001) (Table 3). Segregation index (β = 0.02, 95% CI, −0.03 to 0.06; *P* = .53), children in a single-parent household (β = −0.04, 95% CI, −0.11 to 0.04; *P* = .28), violent crime rate (β = 0.0001; 95% CI, −0.002 to 0.002; *P* = .74), and Gini coefficient (β = −1.02; 95% CI −11.97 to 9.93; *P* = .93) were not associated with MSE incidence on linear regression.

**Table 3.** Factors Independently Associated With Mass Shooting Events^a^

Factor	Standardized β (95% CI)	OR (95% CI)	*P* value
Segregation index	0.02 (−0.03 to 0.06)	1.02 (0.97-1.06)	.53
Children in a single-parent home	−0.04 (−0.11 to 0.04)	0.96 (0.90-1.04)	.28
Violent crime rate	<0.001 (−0.00 to 0.00)	1.00 (1.00-1.00)	.74
Percentage of Black population in MSA	0.10 (0.05 to 0.14)	1.10 (1.05-1.15)	<.001
Gini index	−1.02 (−11.97 to 9.93)	0.36 (0.00-2057.34)	.93

Abbreviations: MSA, metropolitan statistical area; OR, odds ratio.

^a^ Linear regression for factors independently associated with mass shooting events per 100 000. Mean-centered linear regression analysis was applied using best subsets regression: *R* = 0.74, *R^2^* = 0.55.

A separate linear regression was also performed to examine the association between structural racism and the number of people injured in MSEs (eTable 1 in Supplement 1) and then the number of people killed in MSEs (eTable 2 in Supplement 1). The percentage of MSA population that is Black (β = 0.42; 95% CI, 0.23-0.62; *P* < .001) was found to be associated with the number of people injured in MSEs. To examine whether structural racism was associated with the number of deaths in MSEs, linear regression using the same covariates was performed (eTable 2 in Supplement 1). The percentage of MSA population that is Black was again found to be associated with the number of deaths in MSEs (β = 0.08; 95% CI, 0.03-0.13; *P* < .001).

## Discussion

Incidents of mass shootings in which schools or large social gatherings are targeted, such as the Las Vegas shooting in 2017 (60 killed, 413 injured), Pittsburgh Tree of Life Synagogue shooting in 2018 (11 killed, 6 injured), and elementary school shooting in Uvalde in 2022 (22 killed, 18 injured), are high profile and galvanize media attention, but the root causes of more common MSEs are poorly understood. In this study, we sought to examine how measures of structural racism are associated with MSEs to better understand the increasing rates of this form of gun violence. We found that MSA populations with higher percentages of Black individuals were more likely to be affected by MSEs.

Our linear regression showed that cities with a higher Black population are more likely to experience MSEs resulting in more injured and killed individuals than communities with higher White populations. This finding supports that of other studies that used MSE databases and also showed that racial and ethnic minority populations are significantly more likely to be victims of MSEs.^12^ These findings are consistent with rates of community gun violence, which disproportionately affect Black individuals.^5,13^ Beard et al^8^ showed that Black residents in Philadelphia have a firearm assault rate that is 5 times that of their White counterparts. This higher firearm injury rate persists even after correcting for income levels. In fact, the rate of gun violence among the highest income levels in Philadelphia was 15.8 times higher for Black residents than White.^7^ The reasons behind these findings are likely multifactorial and need further investigation. A potential explanation may be related to housing policies, as a long history of redlining has resulted in a higher density of Black residents in certain neighborhoods.^14^ Results from our study, along with data from Beard et al,^8^ suggest the sources of systemic and structural racism that may be associated with MSEs are multifactorial but are rooted in the segregation of neighborhoods or other discriminatory practices related to housing.

Further demonstrating a racial disparity found in major cities, the present study found that not only are Black individuals disproportionately affected by MSEs, but also there are more people injured (eTable 1 in Supplement 1) and more fatalities (eTable 2 in Supplement 1) when the MSEs occur. The reason for these findings could not be examined in this study; however, previous studies have shown that racial disparities exist in the care of injured trauma patients, which may partly explain why the percentage of Black population is associated with increased fatalities in MSEs.^15,16,17^ Trauma deserts, or urban communities farther than 5 miles away from trauma centers, are also more likely to be communities of color, which may contribute to this disparity.^18,19^ Future research must examine how structural factors contribute to the greater loss of life for MSEs in those MSAs with higher percentages of Black individuals.

Although our study did not find an association between income and MSEs, prior studies suggest that income inequality contributes to MSE incidence. In a study by Cabrera and Kwon,^12^ which used the Stanford Mass Shootings of America data project, the authors demonstrated that inequality or income alone can predict MSEs, and together they interact to suggest a stronger correlation. Interestingly, our study found that the Gini coefficient, a measure of income inequality, was not associated with MSE incidence on linear regression. Further research may better define how income inequality and poverty influence the occurrence of MSEs.

While our study focused on the association between factors related to structural racism and mass shootings, statewide firearm legislation cannot be ignored and could be postulated to play a role in MSEs. Interestingly, we noted that Chicago, which had the greatest number of MSEs with a total of 141 events, has a high state firearm legislation grade of an A−. However, there is evidence that demonstrates neighboring state gun laws play a role in a state and city’s gun violence that could be playing a role in Chicago’s high number of MSEs.^20^ In a study by Jewett et al,^21^ the authors analyzed 104 mass public shootings, which they defined as those with at least 4 fatalities. These authors found that 49% were committed by White perpetrators compared with 19% by Black perpetrators.^21^ These data, while important, are difficult to interpret completely because not all cases are resolved with perpetrators identified. In those shootings, reported by Jewett et al,^21^ the majority of guns were legally purchased high-powered firearms, suggesting that state gun laws could play a role in decreasing the incidence.

There was no discernible association noted in this study between gun laws and MSEs with other studies showing similar findings.^22^ However, this study looked at more than 4 years of MSEs during which gun laws varied in strength. Further research could investigate how this has changed throughout the years and whether legislation may influence firearm deaths from MSEs. The overall effect of gun laws on MSE incidence is unclear. In the literature, some evidence shows that the strength of state gun law has no impact on MSE incidence while other studies have shown that tougher gun laws can result in decreased MSE incidence and number of fatalities.^22,23^ Overall, this represents a complex problem that requires further analysis.

### Limitations

There are a number of limitations to this study. As this was a cross-sectional analysis, it is a snapshot in time and therefore limits generalization. Specifically, data were from 2015 to 2019, so they do not reflect the most up-to-date information. However, use of this data set was necessary given the delay in reporting of demographic data and publicly available markers of structural racism. Additionally, analysis during this time period was necessary because data were ascertained from several different sources, given that no centralized database contains all of this information. While some data points were available for MSAs, other pieces of information applied only to the principal city within each MSA. Furthermore, the definition of an MSE can vary depending on the database or article; while the GVA defines an MSE as a shooting with at least 4 injuries or fatalities, other sources use different criteria. Thus, the variations limit interpretation of other data and generalizations. When examining the database, it also is important to know whether the firearms used to inflict injury were obtained legally or illegally. The GVA, which we used as our source, includes any firearm, including those illegally obtained.

Lastly and most importantly, measures of structural racism used in this study and other peer-reviewed articles are imperfect and cannot fully encompass hundreds of years of structural racism in the United States. Particularly, we acknowledge that percentage of a population comprising Black individuals is an imperfect marker despite its use throughout the literature and observation that it is frequently the consequence of decades of racist practices.^5,24,25,26,27,28,29,30,31,32^ Of note, particularly in context of this study’s analysis of MSEs in major metropolitan areas, a growing body of research shows that racial residential segregation practices are predictive of various types of shootings, in much the same way they predict poorer health outcomes, and are independent of other factors such as economic status and education.^8,31,33,34^ As discussed above, previous research has shown that other factors play a role, such as Black-White segregation index, but in our analysis, these were not significant. This is likely because of the complex relationships that exist in dynamic environments and urban communities that drive these MSEs. While firearm homicide mortality has previously been shown to be related to structural racism, the relationship between these various markers to MSEs has not been demonstrated.^5^ In their study of the relationship between structural racism and gun violence, Houghton et al^5^ identified a significant association between markers of structural racism, such as Black-White segregation index and gun violence, that was not noted in the results of our study. One potential explanation is that MSEs represent a different category of violence as compared with community gun violence, and their association with measures of structural racism are not as strong. However, the strong association between race and MSEs shown in our results, viewed in light of other studies in the scientific literature, show that structural racism may play a role in MSEs.^35^ Review of the literature shows that there has been increasing attention to collecting and examining qualitative and quantitative markers of structural racism that can be accurately used in research, particularly studies related to MSEs.^35,36^ Future research is needed to develop more specific and sensitive markers of structural racism: in many of these studies, only a single or few measures of structural racism were found to be significant, not all of them, which suggests the measures are imperfect.^8,12,33^

## Conclusions

This study found that MSEs disproportionately affect Black individuals in major US metropolitan areas, leading to morbidity and mortality. Structural racism appears to be associated with MSE incidence. Public health interventions targeting MSEs must recognize and address systemic inequities that lead to all types of gun violence in the United States.

## Notes

Supplement 1. **eTable 1.** Factors Independently Associated With Number of People Injured in Mass Shooting Events **eTable 2.** Factors Independently Associated With Number of Deaths in Mass Shooting Events

Supplement 2. Data sharing statement
