# Supplementary material for: Association Between Markers of Structural Racism and Mass Shooting Events in Major US Cities
Source: JAMA Surg. 2023 Jul 19;158(10):1032–9. doi: 10.1001/jamasurg.2023.2846 (PMC10357360; doi:10.1001/jamasurg.2023.2846)
Supplement: Supplement 1. — eTable 1. Factors Independently Associated With Number of People Injured in Mass Shooting Events eTable 2. Factors Independently Associated With Number of Deaths in Mass Shooting Events [file jamasurg-e232846-s001.pdf]

Supplemental Online Content

Ghio M, Simpson JT, Ali A, et al. Association between markers of structural racism and mass shooting events in major US cities. *JAMA Surg*. Published online July 19, 2023. doi:10.1001/jamasurg.2023.2846

eTable 1. Factors Independently Associated With Number of People Injured in Mass Shooting Events

eTable 2. Factors Independently Associated With Number of Deaths in Mass Shooting Events

This supplemental material has been provided by the authors to give readers additional information about their work.

© 2023 Ghio M et al. *JAMA Surgery.*

eTable 1. Factors Independently Associated With Number of People Injured in Mass Shooting Events

			95% Confidence Interval		
Independent Factors	Standardized β	OR	Lower Limit	Upper Limit	*p*
Segregation Index	0.012	1.01	-0.206	0.231	0.91
Children in Single Parent Home	-0.121	0.89	-0.439	0.196	0.44
Violent Crime Rate	0.001	1.00	-0.010	0.011	0.95
% African American/Black	0.420	1.52	0.226	0.615	< 0.001
GINI	1.81	3.26	-96.99	100.61	0.97
Mean-centered linear regression analysis was applied using best subsets regression.<br>R=0.73, R2 = 0.53					

Linear regression for factors independently associated with injuries in mass shooting events per 100,000.

© 2023 Ghio M et al. *JAMA Surgery.*

eTable 2. Factors Independently Associated With Number of Deaths in Mass Shooting Events

			95% Confidence Interval		
Independent Factors	Standardized β	OR	Lower Limit	Upper Limit	*p*
Segregation Index	-0.002	1.00	-0.059	0.054	0.93
Children in Single Parent Home	-0.020	0.98	-0.102	0.062	0.62
Violent Crime Rate	0.001	1.00	-0.002	0.003	0.65
% African American/Black	0.076	1.08	0.026	0.126	0.004
GINI	0.638	1.89	-24.85	26.13	0.96
Mean-centered linear regression analysis was applied using best subsets regression.<br>R=0.62, R2 = 0.38					

Linear regression for factors independently associated with mortality in mass shooting events per 100,000.

© 2023 Ghio M et al. *JAMA Surgery.*
