# Supplementary material for: Association Between Markers of Structural Racism and Mass Shooting Events in Major US Cities
Source: JAMA Surg. 2023 Jul 19;158(10):1032–9. doi: 10.1001/jamasurg.2023.2846 (PMC10357360; doi:10.1001/jamasurg.2023.2846)
Supplement: Supplement 2. — Data sharing statement [file jamasurg-e232846-s002.pdf]

Data Sharing Statement

Data
Data available: No

Additional Information
Explanation for why data not available: All data ascertained for this study is publicly available.
